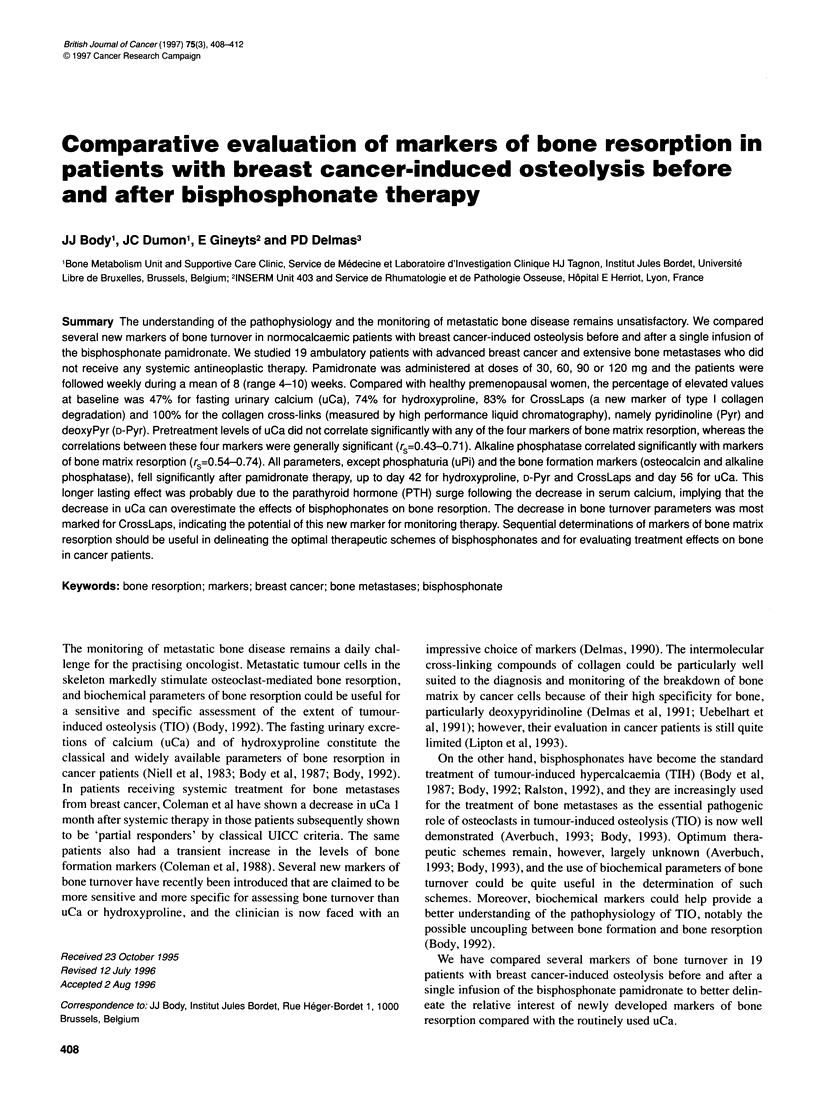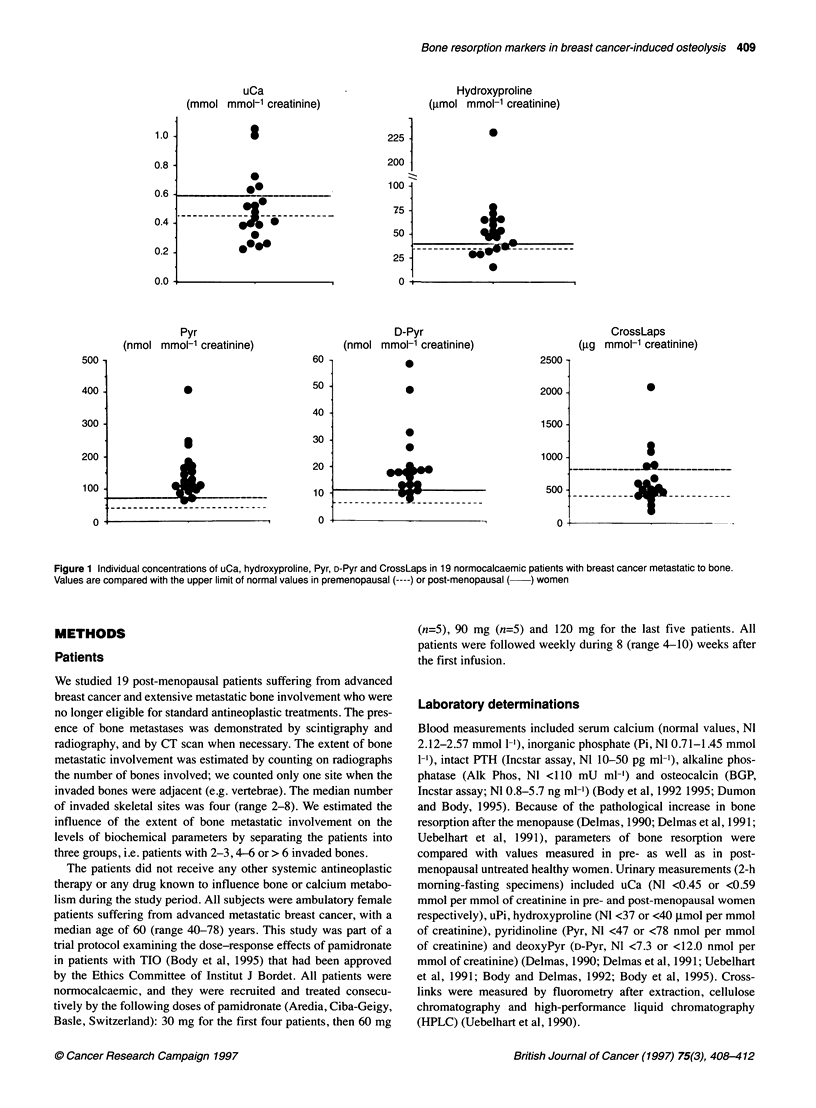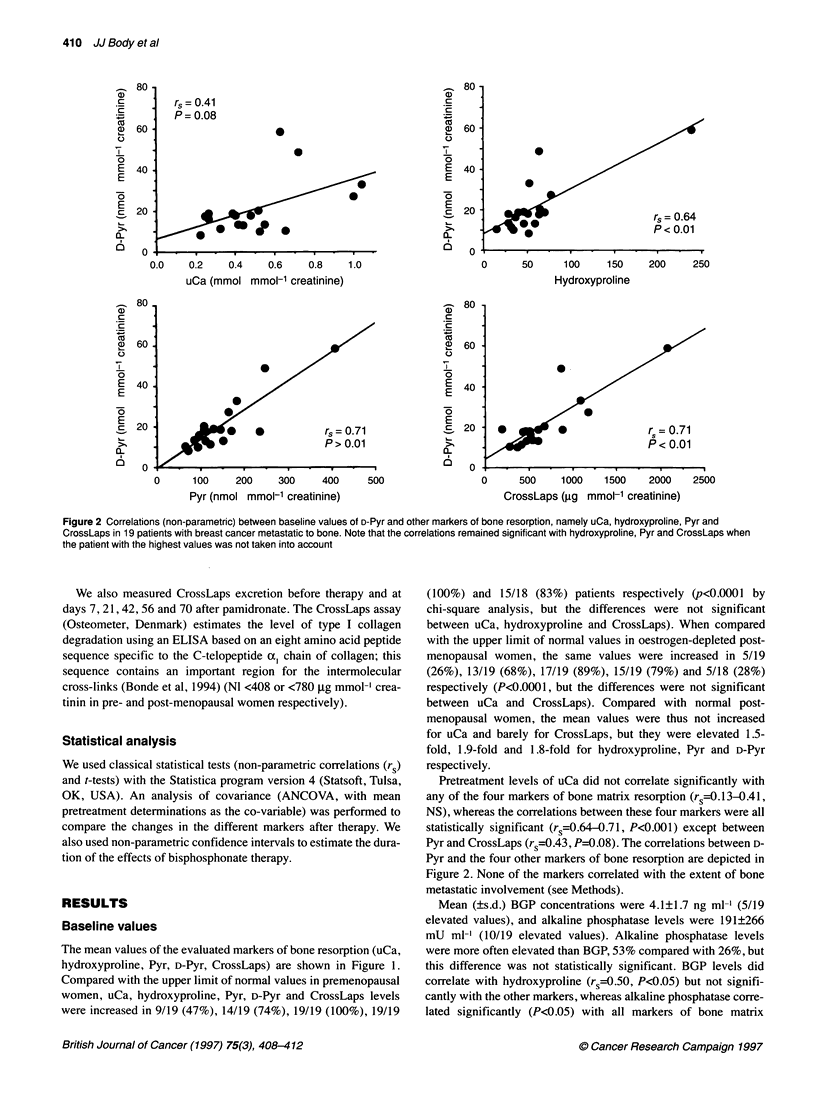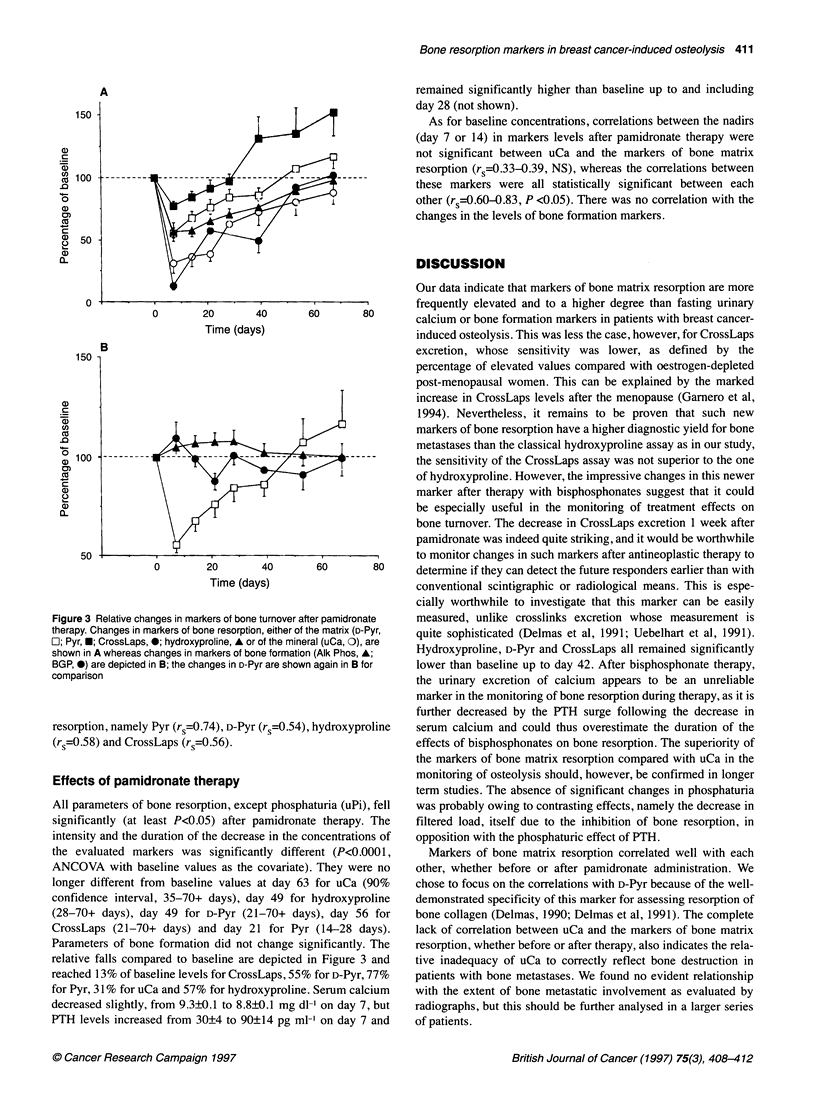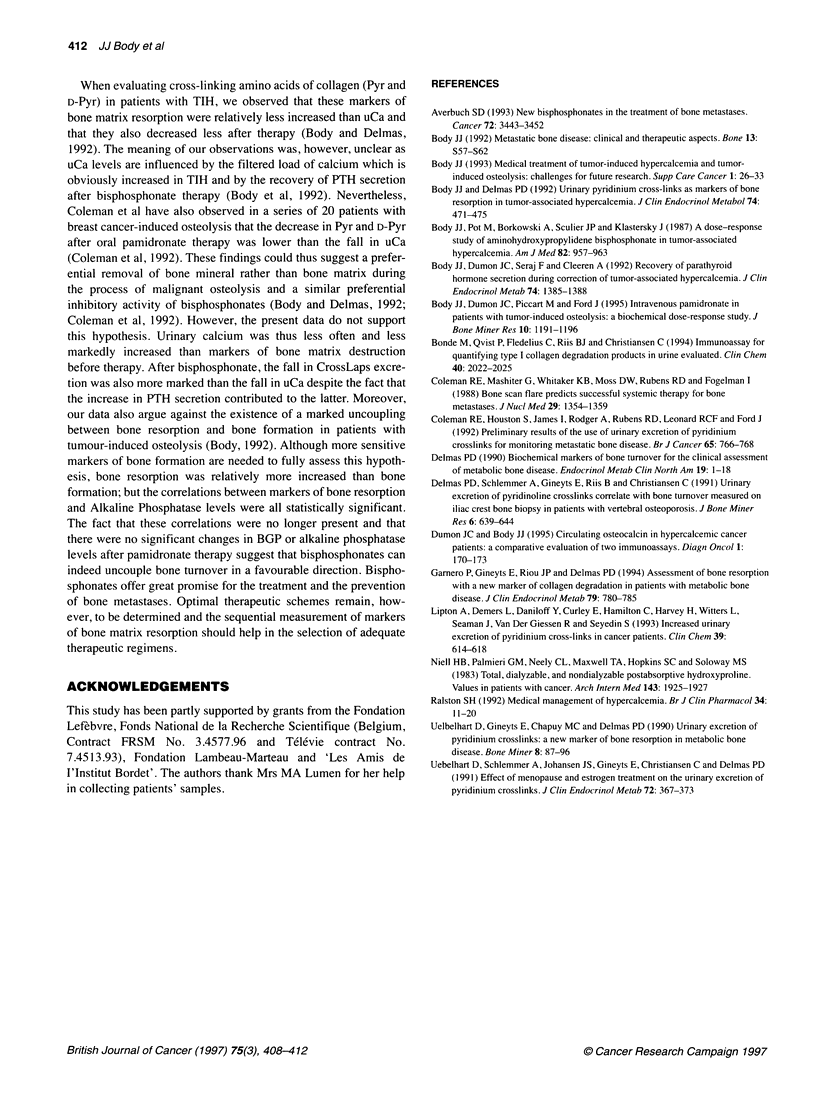# Comparative evaluation of markers of bone resorption in patients with breast cancer-induced osteolysis before and after bisphosphonate therapy.

**DOI:** 10.1038/bjc.1997.66

**Published:** 1997

**Authors:** J. J. Body, J. C. Dumon, E. Gineyts, P. D. Delmas

**Affiliations:** Bone Metabolism Unit and Supportive Care Clinic, Service de Médecine et Laboratoire d'Investigation Clinique HJ Tagnon, Institut Jules Bordet, Université Libre de Bruxelles, Brussels, Belgium.

## Abstract

The understanding of the pathophysiology and the monitoring of metastatic bone disease remains unsatisfactory. We compared several new markers of bone turnover in normocalcaemic patients with breast cancer-induced osteolysis before and after a single infusion of the bisphosphonate pamidronate. We studied 19 ambulatory patients with advanced breast cancer and extensive bone metastases who did not receive any systemic antineoplastic therapy. Pamidronate was administered at doses of 30, 60, 90 or 120 mg and the patients were followed weekly during a mean of 8 (range 4-10) weeks. Compared with healthy premenopausal women, the percentage of elevated values at baseline was 47% for fasting urinary calcium (uCa), 74% for hydroxyproline, 83% for CrossLaps (a new marker of type I collagen degradation) and 100% for the collagen cross-links (measured by high performance liquid chromatography), namely pyridinoline (Pyr) and deoxyPyr (D-Pyr). Pretreatment levels of uCa did not correlate significantly with any of the four markers of bone matrix resorption, whereas the correlations between these four markers were generally significant (r(s)=0.43-0.71). Alkaline phosphatase correlated significantly with markers of bone matrix resorption (r(s)=0.54-0.74). All parameters, except phosphaturia (uPi) and the bone formation markers (osteocalcin and alkaline phosphatase), fell significantly after pamidronate therapy, up to day 42 for hydroxyproline, D-Pyr and CrossLaps and day 56 for uCa. This longer lasting effect was probably due to the parathyroid hormone (PTH) surge following the decrease in serum calcium, implying that the decrease in uCa can overestimate the effects of bisphophonates on bone resorption. The decrease in bone turnover parameters was most marked for CrossLaps, indicating the potential of this new marker for monitoring therapy. Sequential determinations of markers of bone matrix resorption should be useful in delineating the optimal therapeutic schemes of bisphosphonates and for evaluating treatment effects on bone in cancer patients.


British Journal of Cancer (1997) 75(3), 408–412
© 1997 Cancer Research Campaign

### Comparative evaluation of markers of bone resorption in patients with breast cancer-induced osteolysis before and after bisphosphonate therapy

**JJ Body[1], JC Dumon[1], E Gineyts[2] and PD Delmas[3]**

[1]Bone Metabolism Unit and Supportive Care Clinic, Service de Médecine et Laboratoire d'Investigation Clinique HJ Tagnon, Institut Jules Bordet, Université Libre de Bruxelles, Brussels, Belgium; [2]INSERM Unit 403 and Service de Rhumatologie et de Pathologie Osseuse, Hôpital E Herriot, Lyon, France

**Summary** The understanding of the pathophysiology and the monitoring of metastatic bone disease remains unsatisfactory. We compared several new markers of bone turnover in normocalcaemic patients with breast cancer-induced osteolysis before and after a single infusion of the bisphosphonate pamidronate. We studied 19 ambulatory patients with advanced breast cancer and extensive bone metastases who did not receive any systemic antineoplastic therapy. Pamidronate was administered at doses of 30, 60, 90 or 120 mg and the patients were followed weekly during a mean of 8 (range 4–10) weeks. Compared with healthy premenopausal women, the percentage of elevated values at baseline was 47% for fasting urinary calcium (uCa), 74% for hydroxyproline, 83% for CrossLaps (a new marker of type I collagen degradation) and 100% for the collagen cross-links (measured by high performance liquid chromatography), namely pyridinoline (Pyr) and deoxyPyr (D-Pyr). Pretreatment levels of uCa did not correlate significantly with any of the four markers of bone matrix resorption, whereas the correlations between these four markers were generally significant ($r_S$=0.43–0.71). Alkaline phosphatase correlated significantly with markers of bone matrix resorption ($r_S$=0.54–0.74). All parameters, except phosphaturia (uPi) and the bone formation markers (osteocalcin and alkaline phosphatase), fell significantly after pamidronate therapy, up to day 42 for hydroxyproline, D-Pyr and CrossLaps and day 56 for uCa. This longer lasting effect was probably due to the parathyroid hormone (PTH) surge following the decrease in serum calcium, implying that the decrease in uCa can overestimate the effects of bisphophonates on bone resorption. The decrease in bone turnover parameters was most marked for CrossLaps, indicating the potential of this new marker for monitoring therapy. Sequential determinations of markers of bone matrix resorption should be useful in delineating the optimal therapeutic schemes of bisphosphonates and for evaluating treatment effects on bone in cancer patients.

**Keywords:** bone resorption; markers; breast cancer; bone metastases; bisphosphonate

The monitoring of metastatic bone disease remains a daily challenge for the practising oncologist. Metastatic tumour cells in the skeleton markedly stimulate osteoclast-mediated bone resorption, and biochemical parameters of bone resorption could be useful for a sensitive and specific assessment of the extent of tumour-induced osteolysis (TIO) (Body, 1992). The fasting urinary excretions of calcium (uCa) and of hydroxyproline constitute the classical and widely available parameters of bone resorption in cancer patients (Niell et al, 1983; Body et al, 1987; Body, 1992). In patients receiving systemic treatment for bone metastases from breast cancer, Coleman et al have shown a decrease in uCa 1 month after systemic therapy in those patients subsequently shown to be 'partial responders' by classical UICC criteria. The same patients also had a transient increase in the levels of bone formation markers (Coleman et al, 1988). Several new markers of bone turnover have recently been introduced that are claimed to be more sensitive and more specific for assessing bone turnover than uCa or hydroxyproline, and the clinician is now faced with an impressive choice of markers (Delmas, 1990). The intermolecular cross-linking compounds of collagen could be particularly well suited to the diagnosis and monitoring of the breakdown of bone matrix by cancer cells because of their high specificity for bone, particularly deoxypyridinoline (Delmas et al, 1991; Uebelhart et al, 1991); however, their evaluation in cancer patients is still quite limited (Lipton et al, 1993).

On the other hand, bisphosphonates have become the standard treatment of tumour-induced hypercalcaemia (TIH) (Body et al, 1987; Body, 1992; Ralston, 1992), and they are increasingly used for the treatment of bone metastases as the essential pathogenic role of osteoclasts in tumour-induced osteolysis (TIO) is now well demonstrated (Averbuch, 1993; Body, 1993). Optimum therapeutic schemes remain, however, largely unknown (Averbuch, 1993; Body, 1993), and the use of biochemical parameters of bone turnover could be quite useful in the determination of such schemes. Moreover, biochemical markers could help provide a better understanding of the pathophysiology of TIO, notably the possible uncoupling between bone formation and bone resorption (Body, 1992).

We have compared several markers of bone turnover in 19 patients with breast cancer-induced osteolysis before and after a single infusion of the bisphosphonate pamidronate to better delineate the relative interest of newly developed markers of bone resorption compared with the routinely used uCa.

*Received 23 October 1995*
*Revised 12 July 1996*
*Accepted 2 Aug 1996*

*Correspondence to:* JJ Body, Institut Jules Bordet, Rue Héger-Bordet 1, 1000 Brussels, Belgium

**Figure 1** Individual concentrations of uCa, hydroxyproline, Pyr, D-Pyr and CrossLaps in 19 normocalcaemic patients with breast cancer metastatic to bone. Values are compared with the upper limit of normal values in premenopausal (----) or post-menopausal (——) women

### METHODS

### Patients

We studied 19 post-menopausal patients suffering from advanced breast cancer and extensive metastatic bone involvement who were no longer eligible for standard antineoplastic treatments. The presence of bone metastases was demonstrated by scintigraphy and radiography, and by CT scan when necessary. The extent of bone metastatic involvement was estimated by counting on radiographs the number of bones involved; we counted only one site when the invaded bones were adjacent (e.g. vertebrae). The median number of invaded skeletal sites was four (range 2–8). We estimated the influence of the extent of bone metastatic involvement on the levels of biochemical parameters by separating the patients into three groups, i.e. patients with 2–3, 4–6 or > 6 invaded bones.

The patients did not receive any other systemic antineoplastic therapy or any drug known to influence bone or calcium metabolism during the study period. All subjects were ambulatory female patients suffering from advanced metastatic breast cancer, with a median age of 60 (range 40–78) years. This study was part of a trial protocol examining the dose–response effects of pamidronate in patients with TIO (Body et al, 1995) that had been approved by the Ethics Committee of Institut J Bordet. All patients were normocalcaemic, and they were recruited and treated consecutively by the following doses of pamidronate (Aredia, Ciba-Geigy, Basle, Switzerland): 30 mg for the first four patients, then 60 mg ($n$=5), 90 mg ($n$=5) and 120 mg for the last five patients. All patients were followed weekly during 8 (range 4–10) weeks after the first infusion.

### Laboratory determinations

Blood measurements included serum calcium (normal values, Nl 2.12–2.57 mmol $l^{-1}$), inorganic phosphate (Pi, Nl 0.71–1.45 mmol $l^{-1}$), intact PTH (Incstar assay, Nl 10–50 pg $ml^{-1}$), alkaline phosphatase (Alk Phos, Nl <110 mU $ml^{-1}$) and osteocalcin (BGP, Incstar assay; Nl 0.8–5.7 ng $ml^{-1}$) (Body et al, 1992 1995; Dumon and Body, 1995). Because of the pathological increase in bone resorption after the menopause (Delmas, 1990; Delmas et al, 1991; Uebelhart et al, 1991), parameters of bone resorption were compared with values measured in pre- as well as in post-menopausal untreated healthy women. Urinary measurements (2-h morning-fasting specimens) included uCa (Nl <0.45 or <0.59 mmol per mmol of creatinine in pre- and post-menopausal women respectively), uPi, hydroxyproline (Nl <37 or <40 µmol per mmol of creatinine), pyridinoline (Pyr, Nl <47 or <78 nmol per mmol of creatinine) and deoxyPyr (D-Pyr, Nl <7.3 or <12.0 nmol per mmol of creatinine) (Delmas, 1990; Delmas et al, 1991; Uebelhart et al, 1991; Body and Delmas, 1992; Body et al, 1995). Crosslinks were measured by fluorometry after extraction, cellulose chromatography and high-performance liquid chromatography (HPLC) (Uebelhart et al, 1990).

© Cancer Research Campaign 1997

**Figure 2** Correlations (non-parametric) between baseline values of D-Pyr and other markers of bone resorption, namely uCa, hydroxyproline, Pyr and CrossLaps in 19 patients with breast cancer metastatic to bone. Note that the correlations remained significant with hydroxyproline, Pyr and CrossLaps when the patient with the highest values was not taken into account

We also measured CrossLaps excretion before therapy and at days 7, 21, 42, 56 and 70 after pamidronate. The CrossLaps assay (Osteometer, Denmark) estimates the level of type I collagen degradation using an ELISA based on an eight amino acid peptide sequence specific to the C-telopeptide $\alpha_1$ chain of collagen; this sequence contains an important region for the intermolecular cross-links (Bonde et al, 1994) (Nl <408 or <780 μg mmol$^{-1}$ creatinin in pre- and post-menopausal women respectively).

### Statistical analysis

We used classical statistical tests (non-parametric correlations ($r_s$) and $t$-tests) with the Statistica program version 4 (Statsoft, Tulsa, OK, USA). An analysis of covariance (ANCOVA, with mean pretreatment determinations as the co-variable) was performed to compare the changes in the different markers after therapy. We also used non-parametric confidence intervals to estimate the duration of the effects of bisphosphonate therapy.

### RESULTS

### Baseline values

The mean values of the evaluated markers of bone resorption (uCa, hydroxyproline, Pyr, D-Pyr, CrossLaps) are shown in Figure 1. Compared with the upper limit of normal values in premenopausal women, uCa, hydroxyproline, Pyr, D-Pyr and CrossLaps levels were increased in 9/19 (47%), 14/19 (74%), 19/19 (100%), 19/19 (100%) and 15/18 (83%) patients respectively ($p$<0.0001 by chi-square analysis, but the differences were not significant between uCa, hydroxyproline and CrossLaps). When compared with the upper limit of normal values in oestrogen-depleted postmenopausal women, the same values were increased in 5/19 (26%), 13/19 (68%), 17/19 (89%), 15/19 (79%) and 5/18 (28%) respectively ($P$<0.0001, but the differences were not significant between uCa and CrossLaps). Compared with normal postmenopausal women, the mean values were thus not increased for uCa and barely for CrossLaps, but they were elevated 1.5-fold, 1.9-fold and 1.8-fold for hydroxyproline, Pyr and D-Pyr respectively.

Pretreatment levels of uCa did not correlate significantly with any of the four markers of bone matrix resorption ($r_s$=0.13–0.41, NS), whereas the correlations between these four markers were all statistically significant ($r_s$=0.64–0.71, $P$<0.001) except between Pyr and CrossLaps ($r_s$=0.43, $P$=0.08). The correlations between D-Pyr and the four other markers of bone resorption are depicted in Figure 2. None of the markers correlated with the extent of bone metastatic involvement (see Methods).

Mean (±s.d.) BGP concentrations were 4.1±1.7 ng ml$^{-1}$ (5/19 elevated values), and alkaline phosphatase levels were 191±266 mU ml$^{-1}$ (10/19 elevated values). Alkaline phosphatase levels were more often elevated than BGP, 53% compared with 26%, but this difference was not statistically significant. BGP levels did correlate with hydroxyproline ($r_s$=0.50, $P$<0.05) but not significantly with the other markers, whereas alkaline phosphatase correlated significantly ($P$<0.05) with all markers of bone matrix

© Cancer Research Campaign 1997

**Figure 3** Relative changes in markers of bone turnover after pamidronate therapy. Changes in markers of bone resorption, either of the matrix (D-Pyr, □; Pyr, ■; CrossLaps, ●; hydroxyproline, ▲ or of the mineral (uCa, ○), are shown in **A** whereas changes in markers of bone formation (Alk Phos, ▲; BGP, ●) are depicted in **B**; the changes in D-Pyr are shown again in **B** for comparison resorption, namely Pyr ($r_S$=0.74), D-Pyr ($r_S$=0.54), hydroxyproline ($r_S$=0.58) and CrossLaps ($r_S$=0.56).

### Effects of pamidronate therapy

All parameters of bone resorption, except phosphaturia (uPi), fell significantly (at least $P$<0.05) after pamidronate therapy. The intensity and the duration of the decrease in the concentrations of the evaluated markers was significantly different ($P$<0.0001, ANCOVA with baseline values as the covariate). They were no longer different from baseline values at day 63 for uCa (90% confidence interval, 35–70+ days), day 49 for hydroxyproline (28–70+ days), day 49 for D-Pyr (21–70+ days), day 56 for CrossLaps (21–70+ days) and day 21 for Pyr (14–28 days). Parameters of bone formation did not change significantly. The relative falls compared to baseline are depicted in Figure 3 and reached 13% of baseline levels for CrossLaps, 55% for D-Pyr, 77% for Pyr, 31% for uCa and 57% for hydroxyproline. Serum calcium decreased slightly, from 9.3±0.1 to 8.8±0.1 mg $dl^{-1}$ on day 7, but PTH levels increased from 30±4 to 90±14 pg $ml^{-1}$ on day 7 and remained significantly higher than baseline up to and including day 28 (not shown).

As for baseline concentrations, correlations between the nadirs (day 7 or 14) in markers levels after pamidronate therapy were not significant between uCa and the markers of bone matrix resorption ($r_S$=0.33–0.39, NS), whereas the correlations between these markers were all statistically significant between each other ($r_S$=0.60–0.83, $P$ <0.05). There was no correlation with the changes in the levels of bone formation markers.

### DISCUSSION

Our data indicate that markers of bone matrix resorption are more frequently elevated and to a higher degree than fasting urinary calcium or bone formation markers in patients with breast cancer-induced osteolysis. This was less the case, however, for CrossLaps excretion, whose sensitivity was lower, as defined by the percentage of elevated values compared with oestrogen-depleted post-menopausal women. This can be explained by the marked increase in CrossLaps levels after the menopause (Garnero et al, 1994). Nevertheless, it remains to be proven that such new markers of bone resorption have a higher diagnostic yield for bone metastases than the classical hydroxyproline assay as in our study, the sensitivity of the CrossLaps assay was not superior to the one of hydroxyproline. However, the impressive changes in this newer marker after therapy with bisphosphonates suggest that it could be especially useful in the monitoring of treatment effects on bone turnover. The decrease in CrossLaps excretion 1 week after pamidronate was indeed quite striking, and it would be worthwhile to monitor changes in such markers after antineoplastic therapy to determine if they can detect the future responders earlier than with conventional scintigraphic or radiological means. This is especially worthwhile to investigate that this marker can be easily measured, unlike crosslinks excretion whose measurement is quite sophisticated (Delmas et al, 1991; Uebelhart et al, 1991). Hydroxyproline, D-Pyr and CrossLaps all remained significantly lower than baseline up to day 42. After bisphosphonate therapy, the urinary excretion of calcium appears to be an unreliable marker in the monitoring of bone resorption during therapy, as it is further decreased by the PTH surge following the decrease in serum calcium and could thus overestimate the duration of the effects of bisphosphonates on bone resorption. The superiority of the markers of bone matrix resorption compared with uCa in the monitoring of osteolysis should, however, be confirmed in longer term studies. The absence of significant changes in phosphaturia was probably owing to contrasting effects, namely the decrease in filtered load, itself due to the inhibition of bone resorption, in opposition with the phosphaturic effect of PTH.

Markers of bone matrix resorption correlated well with each other, whether before or after pamidronate administration. We chose to focus on the correlations with D-Pyr because of the well-demonstrated specificity of this marker for assessing resorption of bone collagen (Delmas, 1990; Delmas et al, 1991). The complete lack of correlation between uCa and the markers of bone matrix resorption, whether before or after therapy, also indicates the relative inadequacy of uCa to correctly reflect bone destruction in patients with bone metastases. We found no evident relationship with the extent of bone metastatic involvement as evaluated by radiographs, but this should be further analysed in a larger series of patients.

*© Cancer Research Campaign 1997* 

When evaluating cross-linking amino acids of collagen (Pyr and D-Pyr) in patients with TIH, we observed that these markers of bone matrix resorption were relatively less increased than uCa and that they also decreased less after therapy (Body and Delmas, 1992). The meaning of our observations was, however, unclear as uCa levels are influenced by the filtered load of calcium which is obviously increased in TIH and by the recovery of PTH secretion after bisphosphonate therapy (Body et al, 1992). Nevertheless, Coleman et al have also observed in a series of 20 patients with breast cancer-induced osteolysis that the decrease in Pyr and D-Pyr after oral pamidronate therapy was lower than the fall in uCa (Coleman et al, 1992). These findings could thus suggest a preferential removal of bone mineral rather than bone matrix during the process of malignant osteolysis and a similar preferential inhibitory activity of bisphosphonates (Body and Delmas, 1992; Coleman et al, 1992). However, the present data do not support this hypothesis. Urinary calcium was thus less often and less markedly increased than markers of bone matrix destruction before therapy. After bisphosphonate, the fall in CrossLaps excretion was also more marked than the fall in uCa despite the fact that the increase in PTH secretion contributed to the latter. Moreover, our data also argue against the existence of a marked uncoupling between bone resorption and bone formation in patients with tumour-induced osteolysis (Body, 1992). Although more sensitive markers of bone formation are needed to fully assess this hypothesis, bone resorption was relatively more increased than bone formation; but the correlations between markers of bone resorption and Alkaline Phosphatase levels were all statistically significant. The fact that these correlations were no longer present and that there were no significant changes in BGP or alkaline phosphatase levels after pamidronate therapy suggest that bisphosphonates can indeed uncouple bone turnover in a favourable direction. Bisphosphonates offer great promise for the treatment and the prevention of bone metastases. Optimal therapeutic schemes remain, however, to be determined and the sequential measurement of markers of bone matrix resorption should help in the selection of adequate therapeutic regimens.

### ACKNOWLEDGEMENTS

This study has been partly supported by grants from the Fondation Lefèbvre, Fonds National de la Recherche Scientifique (Belgium, Contract FRSM No. 3.4577.96 and Télévie contract No. 7.4513.93), Fondation Lambeau-Marteau and 'Les Amis de l'Institut Bordet'. The authors thank Mrs MA Lumen for her help in collecting patients' samples.

### REFERENCES

Averbuch SD (1993) New bisphosphonates in the treatment of bone metastases. *Cancer* **72**: 3443–3452

Body JJ (1992) Metastatic bone disease: clinical and therapeutic aspects. *Bone* **13**: S57–S62

Body JJ (1993) Medical treatment of tumor-induced hypercalcemia and tumor-induced osteolysis: challenges for future research. *Supp Care Cancer* **1**: 26–33

Body JJ and Delmas PD (1992) Urinary pyridinium cross-links as markers of bone resorption in tumor-associated hypercalcemia. *J Clin Endocrinol Metabol* **74**: 471–475

Body JJ, Pot M, Borkowski A, Sculier JP and Klastersky J (1987) A dose–response study of aminohydroxypropylidene bisphosphonate in tumor-associated hypercalcemia. *Am J Med* **82**: 957–963

Body JJ, Dumon JC, Seraj F and Cleeren A (1992) Recovery of parathyroid hormone secretion during correction of tumor-associated hypercalcemia. *J Clin Endocrinol Metab* **74**: 1385–1388

Body JJ, Dumon JC, Piccart M and Ford J (1995) Intravenous pamidronate in patients with tumor-induced osteolysis: a biochemical dose-response study. *J Bone Miner Res* **10**: 1191–1196

Bonde M, Qvist P, Fledelius C, Riis BJ and Christiansen C (1994) Immunoassay for quantifying type I collagen degradation products in urine evaluated. *Clin Chem* **40**: 2022–2025

Coleman RE, Mashiter G, Whitaker KB, Moss DW, Rubens RD and Fogelman I (1988) Bone scan flare predicts successful systemic therapy for bone metastases. *J Nucl Med* **29**: 1354–1359

Coleman RE, Houston S, James I, Rodger A, Rubens RD, Leonard RCF and Ford J (1992) Preliminary results of the use of urinary excretion of pyridinium crosslinks for monitoring metastatic bone disease. *Br J Cancer* **65**: 766–768

Delmas PD (1990) Biochemical markers of bone turnover for the clinical assessment of metabolic bone disease. *Endocrinol Metab Clin North Am* **19**: 1–18

Delmas PD, Schlemmer A, Gineyts E, Riis B and Christiansen C (1991) Urinary excretion of pyridinoline crosslinks correlate with bone turnover measured on iliac crest bone biopsy in patients with vertebral osteoporosis. *J Bone Miner Res* **6**: 639–644

Dumon JC and Body JJ (1995) Circulating osteocalcin in hypercalcemic cancer patients: a comparative evaluation of two immunoassays. *Diagn Oncol* **1**: 170–173

Garnero P, Gineyts E, Riou JP and Delmas PD (1994) Assessment of bone resorption with a new marker of collagen degradation in patients with metabolic bone disease. *J Clin Endocrinol Metab* **79**: 780–785

Lipton A, Demers L, Daniloff Y, Curley E, Hamilton C, Harvey H, Witters L, Seaman J, Van Der Giessen R and Seyedin S (1993) Increased urinary excretion of pyridinium cross-links in cancer patients. *Clin Chem* **39**: 614–618

Niell HB, Palmieri GM, Neely CL, Maxwell TA, Hopkins SC and Soloway MS (1983) Total, dialyzable, and nondialyzable postabsorptive hydroxyproline. Values in patients with cancer. *Arch Intern Med* **143**: 1925–1927

Ralston SH (1992) Medical management of hypercalcemia. *Br J Clin Pharmacol* **34**: 11–20

Uelbelhart D, Gineyts E, Chapuy MC and Delmas PD (1990) Urinary excretion of pyridinium crosslinks: a new marker of bone resorption in metabolic bone disease. *Bone Miner* **8**: 87–96

Uebelhart D, Schlemmer A, Johansen JS, Gineyts E, Christiansen C and Delmas PD (1991) Effect of menopause and estrogen treatment on the urinary excretion of pyridinium crosslinks. *J Clin Endocrinol Metab* **72**: 367–373

© Cancer Research Campaign 1997